# Improvement in 100-m Sprint Performance at an Altitude of 2250 m

**DOI:** 10.3390/sports4020029

**Published:** 2016-05-12

**Authors:** Nicholas P. Linthorne

**Affiliations:** Department of Life Sciences, Brunel University London, Uxbridge, Middlesex, UB8 3PH, UK; nick.linthorne@brunel.ac.uk; Tel.: +44-01895-266479

**Keywords:** altitude, athletics, Olympic Games, sprinting

## Abstract

A fair system of recognizing records in athletics should consider the influence of environmental conditions on performance. The aim of this study was to determine the effect of an altitude of 2250 m on the time for a 100-m sprint. Competition results from the 13 Olympic Games between 1964 and 2012 were corrected for the effects of wind and de-trended for the historical improvement in performance. The time advantage due to competing at an altitude of 2250 m was calculated from the difference between the mean race time at the 1968 Olympic Games in Mexico City and the mean race times at the low-altitude competition venues. The observed time advantage of Mexico City was 0.19 (±0.02) s for men and 0.21 (±0.05) s for women (±90% confidence interval). These results indicate that 100-m sprinters derive a substantial performance advantage when competing at a high-altitude venue and that an altitude of 1000 m provides an advantage equivalent to a 2 m/s assisting wind (0.10 s). Therefore, the altitude of the competition venue as well as the wind speed during the race should be considered when recognizing record performances.

## 1. Introduction

Although head-to-head competition is the primary purpose of an organized athletics meeting, the setting of record performances is also of considerable interest to athletes and spectators. Performance in a 100-m sprint can be substantially aided by a strong following wind and so the International Association of Athletic Federations (IAAF) has a limit of 2 m/s for the allowable assisting wind when recognizing 100-m records [1]. However, there is currently no restriction on the altitude of the competition venue, even though there is strong empirical evidence that sprint performances are substantially aided by a high-altitude venue [2,3,4,5]. A high-altitude competition venue is believed to improve sprint performance through a reduction in air density, which reduces the aerodynamic drag force acting on the athlete and hence allows the athlete to reach a greater top speed [6]. This benefit is expected to be partly offset by the lower partial pressure of oxygen in the atmosphere, which reduces the aerobic power that can be generated by the athlete’s muscles and, hence, increases the time taken to cover the race distance [7].

To date, there is no firmly accepted value for the effect of altitude on a 100-m sprint performance. Several modelling studies have calculated the time advantage of a performance at an altitude of 2250 m (the altitude of Mexico City, site of the 1968 Olympic Games). However, the results range considerably, from as low as 0.05 s to as high as 0.18 s [7,8,9,10,11,12]. The results from these modelling studies are strongly dependent on the values of aerodynamic and power generation parameters, but some of these parameter values are not particularly well known (e.g., the drag area of the athlete).

Several studies have shown that results from sports competitions can be an extensive source of carefully measured data for use in biomechanical and physiological studies [2,12,13,14]. By selecting appropriate competition data, an investigator can study the influence of a chosen variable on athletic performance. Therefore, one way of quantifying the effect of altitude on 100-m sprint times is to compare competition performances achieved at high-altitude venues to those achieved at venues near to sea-level. Ward-Smith [6] and Behncke [9] conducted this type of study using competition results from the Olympic Games. Ward-Smith found that the men’s 100-m sprint performances at the Mexico City 1968 Olympic Games (altitude 2250 m) were improved by 0.17 (±0.13) s (±90% confidence interval) relative to performances at other Olympic Games, and Behncke calculated that performances were improved by 0.17 (±0.10) s for men and 0.18 (±0.13) s for women. Unfortunately, these studies had large confidence intervals associated with their results because they compared only a few competitions (five and three, respectively) and used performances by only a few athletes in each competition (three and six, respectively). Also, the accuracy of the results is questionable because the studies did not correct the race times for the effect of wind, used a mix of hand times and electronic times, and Behnke did not account for the historical trend in performance.

The aim of the present study was to obtain a more accurate estimate of the effect of altitude on 100-m sprint times by conducting a more substantive analysis of competition results from the Olympic Games. The present study analyzed race times by every 100-m competitor in the 13 Olympic Games competitions between 1964 and 2012. This study had a more thorough account of the confounding factors in the competition data (including the effect of wind and the historical trend in performance), and so was expected to give a more accurate estimate of the time advantage of altitude at the Mexico City 1968 Olympic Games (and with a smaller confidence interval) than those obtained in previous studies.

## 2. Methods

The electronic times and wind readings for all rounds of the 100-m competitions at the Olympic Games between 1964 and 2012 (about 3250 performances) were obtained from the archives of the Association of Track and Field Statisticians (ATFS). For the Olympic Games prior to 1964 the record of electronic times and wind readings is not complete and so these competitions were not included in the study. The possibility of a gender difference in the effect of altitude on 100-m sprint performance was considered by analyzing the men’s and women’s competitions separately.

The time advantage of competing at an altitude of 2250 m was obtained by calculating an average performance at each Olympic Games, plotting the results as a function of the year of competition, and then comparing the result from the Mexico City 1968 competition to the data from the other competitions. To obtain an accurate value for the time advantage of an altitude of 2250 m, the confounding factors in the Olympic Games competition data (both between the competitions and within each competition) needed to be considered. In this study the velocity of the wind during the race, the historical trend in 100-m performances, and the sub-maximal performances in the early rounds of the competition were expected to be the largest confounding factors. Other lesser factors were expected to be the non-zero altitude of some of the competition venues, variations in the standard of the athletes at the competitions, and different types of track surface.

### 2.1. Corrections for Wind and Historical Trend

The competition performances from the Olympic Games were adjusted to a zero wind performance, *T_o_*, using

*T*_o_ = *T* + *α* (*V*_w_ − *βV*_w_^2^), (1)

where *T* is the athlete’s official race time; *V*_w_ is the wind reading; and *α* and *β* are constants [14]. For international standard sprinters *α* = 0.050 s/m, and *β* = 0.0556 s^2^/m for men and *β* = 0.0667 s^2^/m for women. The time advantage of a 2 m/s tailwind is therefore about 0.10 s for men and about 0.12 s for women.

The mathematical form of the historical trend in 100-m performances was determined by examining the world ranking lists for the period between 1950 and 2015. Performances prior to 1975 are hand times and so a correction of 0.24 s was applied to these times [15]. The mean of the twenty highest-ranked athletes in each year was calculated, the mean values were plotted as a function of year, and a selection of mathematical equations was fitted to the data. The fitted curves included a second-order polynomial, a third-order polynomial, a rectangular hyperbola, an exponential curve, a four-parameter symmetric logistic curve, a five-parameter logistic curve, and a six-parameter generalized logistic curve. The decision about the most appropriate curve was guided by a locally weighted regression (loess) fit to the data and by examining the distribution of the residuals [16]. If two or more fitted curves seemed appropriate for the data, a calculation of Akaike’s Information Criterion was used to determine which of the curves gave the best fit [17].

### 2.2. Calculation of Mean Race Time for an Olympic Games Competition

Athletes do not always produce a maximum-effort performance in every round of a 100-m competition [14]. Therefore, for each of the 13 Olympic Games competitions, the athletes’ best wind-corrected performance in the competition was identified and the athletes were ranked according to this performance (*i.e.*, *A*_1_, *A*_2_, *A*_3_, … *A*_n_, for the athletes ranked 1 to *n*, where *n* is the number of athletes in the competition). The mean race time for each Olympic Games competition was then calculated from the ranking list of athletes. However, the uncertainty in a mean race time depends on the number of athletes used in the calculation [18]. In addition, including the highest-ranked athletes (*i.e.*, 1st, 2nd, 3rd, …) in the calculation of the mean race time might not produce the lowest possible uncertainty in the mean race time. To identify the optimum selection of athletes to include in the calculation of the mean race time, the mean race time (and its associated uncertainty) was calculated using (*n*_mean_ =) 5, 10, 15, 20, 25, 30, 35, or 40 athletes, and for each choice of *n*_mean_ the middle-ranked athlete in the group (*A*_mid_) was systematically varied over a wide range. As an illustration of this process, if the calculation of the mean race time used five athletes and the middle-ranked athlete in the group of five was the 27th-ranked athlete (*i.e.*, *n*_mean_ = 5 and *A*_mid_ = 27), then the calculation used athletes *A*_25_, *A*_26_, *A*_27_, *A*_28_, and *A*_29_. For each Olympic Games competition the uncertainty in the mean race time was plotted against *A*_mid_ and *n*_mean_. These plots were examined to identify the optimum *n*_mean_ and *A*_mid_ that minimized the uncertainty in the mean race time. These optimum values were then used to calculate the mean race time for each of the Olympic Games competitions.

### 2.3. Time Advantage of Mexico City

To calculate the time advantage of competing at Mexico City, the historical improvement in performance was removed by de-trending the Olympic Games competition data with a curve of the same shape as the best fit to the world rankings data. The difference of the Mexico City Olympic Games data from the mean time of the other Olympic Games competitions was then taken as the time advantage of competing at an altitude of 2250 m.

### 2.4. Other Confounding Factors

In this study it was initially assumed that all the Olympic Games competitions between 1964 and 2012 (except Mexico City 1968) were held at venues close to sea-level. However, the Munich 1972 and Atlanta 1996 Olympic Games were held at venues that are at a moderately high altitude (Munich, 520 m; Atlanta, 305 m). The time advantages of competing at Munich and Atlanta were expected to be about 30% and 18% of the time advantage of competing at Mexico City [8]. Therefore, corrections of 30% and 18% were applied to the Munich 1972 and Atlanta 1996 data, and the Olympic Games data were re-analyzed. The effects of the altitude of the Munich and Atlanta competition venues were also tested by removing the Munich 1972 and Atlanta 1996 data from the analysis.

The Olympic Games is the world’s premier athletics competition and many of the best 100-m sprinters attended the competitions. However, the 1976 Olympic Games in Montreal was boycotted by 18 nations (mostly African), the 1980 Olympic Games in Moscow was boycotted by many Western nations (most notably USA, Canada, and West Germany), and the 1984 Olympic Games in Los Angeles was boycotted by 17 Eastern Bloc nations (most notably USSR, East Germany, Bulgaria, Poland, and Cuba). The effects of these boycotts were tested by removing the affected competitions and re-analyzing the Olympic Games data.

The Tokyo 1964 Olympic Games was held on a cinders track, but all subsequent Olympic Games competitions were conducted on synthetic tracks. To test for the effects of different types of track surface in the Olympic Games data, the Tokyo 1964 competition was removed and the Olympic Games data were re-analyzed.

## 3. Results

**Figure 1:**
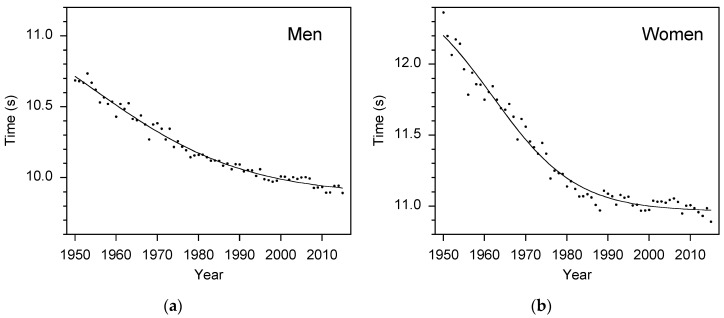
These plots show the historical trend in world 100-m standards; (**a**) men, (**b**) women. Data points are the mean of the top 20 athletes in each year. The fitted curve is a 4-parameter logistic curve (Equation (2)). Coefficient of determination: men *r*^2^ = 0.98; women *r*^2^ = 0.97.

Figure 1 shows the mean of the twenty highest-ranked athletes in each year between 1950 and 2015. The best fit curve for both the men and women was a four-parameter logistic curve [19],

(2) $$T_{\mathrm{rank}}=\frac{T_{\max}-T_{\min}}{1+e^{-a(Y-Y_{\inf})}}+T_{\min},$$

where *T*_rank_ is the mean time of the top 20 athletes in the world rankings; *Y* is the year; *T*_max_ is the asymptotic maximum time; *T*_min_ is the asymptotic minimum time; *Y*_inf_ is the year at the inflection point of the curve; and *a* is a measure of the growth rate. The values of the regression coefficients were *T*_max_ = 11.37 s, *T*_min_ = 9.87 s, *Y*_inf_ = 1955 years, and *a* = –0.054 per year for the men; and *T*_max_ = 12.58 s, *T*_min_ = 10.96 s, *Y*_inf_ = 1962 years, and *a* = –0.098 per year for the women.

**Figure 2:**
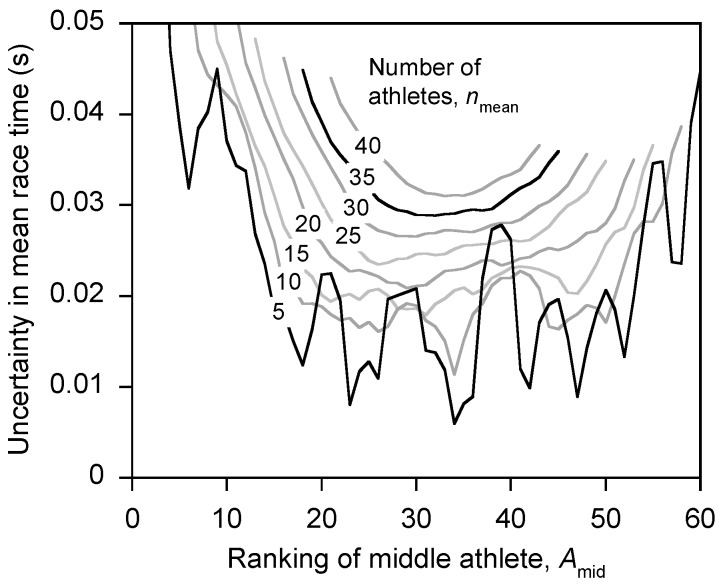
This plot shows there is an optimum choice of athletes to include in the calculation of the mean race time at an Olympic Games competition. The uncertainty (90% confidence interval) in the mean race time is lowest when the ranking of the middle athlete in the group of athletes used in the calculation is about *A*_mid_ = 20–45. When the number of athletes used in the calculation of the mean race time is less than about *n*_mean_ = 20, the uncertainty in the mean value is not reliable (*i.e.*, subject to excessive random fluctuations). The optimum values were taken to be *A*_mid_ = 30 and *n*_mean_ = 20. Data for the men at the Mexico City 1968 Olympic Games.

There was an optimum choice of athletes to include in the calculation of the mean race time for an Olympic Games competition. For the men, the uncertainty (90% confidence limits) in the mean race time was lowest when the middle-ranked athlete in the calculation (*A*_mid_) was ranked about 20–45, and a reliable value of the uncertainty (with only small random fluctuations) was attained when using greater than about 20 athletes in the calculation (Figure 2). Therefore, the mean race time for the Olympic Games competitions was taken as that calculated with *n*_mean_ = 20 and *A*_mid_ = 30. In a similar fashion, for the women the mean race time was taken as that calculated with *n*_mean_ = 20 and *A*_mid_ = 20. Figure 2 highlights that the uncertainty in the mean race time is substantially reduced when the top-ranked athletes in the competition are not included in the calculation.

**Figure 3:**
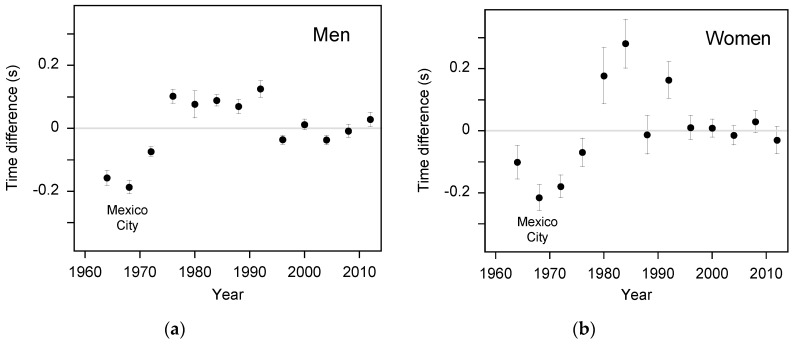
These plots show the mean race times at Olympic Games competitions from 1964 to 2012 after de-trending with a curve of the same shape as the historical trend in 100-m performances; (**a**) men, (**b**) women. Vertical error bars indicate the 90% confidence interval in the mean. There is a substantial deviation at the Mexico City 1968 Olympic Games due to the high altitude of this site (2250 m).

A plot of the mean race time for each Olympic Games competition showed a clear historical trend of improving performances, and this trend was similar to that of the world rankings data. The curve that was fitted to the Olympic Games data was constrained to be the same shape as the historical trend in 100-m performances by setting the values of *T*_max_, *T*_min_, *Y*_inf_, and *a* to be equal to those obtained from the fit to the world rankings data. When calculating the fitted curve, the mean race time at each Olympic Games competition was weighted by the 90% confidence interval in the mean race time. For both the men and women, the deviations of the mean race times at each Olympic Games competition from the fitted curve showed a clear perturbation for the Mexico City 1968 Olympic Games (Figure 3). The time advantage of Mexico City was 0.19 (±0.02) s for the men and 0.22 (±0.05) s for the women.

The time advantage of Mexico City was insensitive to the number of athletes (*n*_mean_) used to calculate the mean race time at the competitions. Using 15 or 25 athletes (rather than 20) changed the calculated time advantage of Mexico City by less than 0.01 s. Similarly, the time advantage of Mexico City was insensitive to the choice of middle-ranked athlete (*A*_mid_). For the men, using the 20th or 40th ranked athlete (rather than the 30th) changed the time advantage by less than 0.01 s. For the women, using the 10th or 30th ranked athlete (rather than the 20th) changed the time advantage by less than 0.01 s.

Applying a 30% altitude correction to the Munich 1972 data (0.05–0.07 s) had little effect on the results. The calculated time advantages of Mexico City were increased by less than 0.01 s and there was little change in the uncertainties. Likewise, applying an 18% altitude correction to the Atlanta 1996 data (0.03–0.04 s) had little effect on the results. Removing the data for the boycotted competitions had only a small effect on the results. With the Moscow 1980 and Los Angeles 1984 competitions removed the calculated time advantages of Mexico City were reduced by about 0.01 s, and with the Montreal 1976 competition removed the calculated time advantages of Mexico City were reduced by less than 0.01 s. When the Tokyo 1964 competition (which was held on a cinders track) was removed from the analysis the calculated time advantages of Mexico City were increased by about 0.01 s.

In summary, the calculated time advantage of Mexico City was insensitive to the choices of *n*_mean_ and *A*_mid_, the non-zero altitude of some of the competition venues, boycotts at competitions, and different types of track surface. For the men, the most accurate estimate from this study is probably that with an altitude correction for the Munich 1972 Olympic Games. For this scenario the time advantage of Mexico City is 0.19 (±0.02) s. For the women, the most accurate estimate from this study is probably that with an altitude correction for Munich 1972 and with the Moscow 1980 and Los Angeles 1984 competitions removed from the data. For this scenario the time advantage of Mexico City is 0.21 (±0.05) s. The uncertainty in the time advantage of Mexico City was mainly due to the uncertainty in the mean race time at the Mexico City 1968 Olympic Games, rather than due to the uncertainty in the curve fitted to the Olympic Games competition data.

## 4. Discussion

This study confirmed that athletes derived a substantial performance advantage in the 100-m at the Mexico City 1968 Olympic Games. The estimates of the time advantage were similar to those obtained by Ward-Smith [6] and Behncke [9]. However, the estimates from the present study have a considerably lower uncertainty, which was mostly due to analyzing a more appropriate selection of athletes. Also, the estimates obtained in the present study should be more accurate because the race times were corrected for the effect of wind and for the historical trend in performances.

The results from the present study indicate that there is no substantial difference between men and women in the time advantage of competing at a high-altitude venue. This finding is consistent with results from mathematical modelling studies [7,8], which predict only small gender differences (less than 0.01 s) due to the combined effects of differences in body size, the time to complete the race, and the athlete’s velocity profile.

The uncertainty in the calculated time advantage of Mexico City reported for this study is purely statistical and does not include systematic factors. Although considerable care was taken to account for the most likely confounding factors (*i.e.*, wind velocity, the historical trend in performance, submaximal performances in the early rounds, variations in the standard of athletes at the competition, and track surface), Figure 3 shows there is still substantial unexplained variance in the mean race times for the Olympic Games competitions. That is, there might be other confounding factors that were not considered in this study. Potential factors include: the time of year when the competition was held; differences in the time for the sound of the starting gun to travel to the athletes from the starting gun; differences in athlete footwear and track stiffness; inter-athlete differences in frontal area; the effects of differences in air temperature, barometric pressure, and humidity on the aerodynamic drag experienced by an athlete [6,20]; and the effect of temperature on the generation of muscular power. We cannot exclude the possibility that one or more of these factors (or another unidentified factor) made a substantial contribution to the calculated time advantage of the 1968 Mexico City Olympic Games.

### 4.1. Other Competition Analyses and Mathematical Models

Hollings and colleagues [2] also used competition data to determine the effect of altitude on 100-m sprint times. However, they did not use data from the Mexico City 1968 Olympic Games. Rather, they used mixed linear modelling to examine performances by elite male athletes in major competitions (6628 performances by 56 athletes) during a ten-year period from 2000 to 2009. They found that performances produced at venues above 1000 m were 0.05 (±0.02) s (±99% confidence interval) faster than those produced at venues below 1000 m. Assuming an average altitude difference of 1000 m between the two groups of performances, and assuming a relation between altitude and time advantage proposed by Arsac [8], the result from Hollings and colleagues indicates that competing at Mexico City (2250 m) should confer a time advantage of about 0.10 (±0.04) s. The accuracy of the altitude result obtained by Hollings and colleagues is strengthened by their result for the effect of a 2 m/s tailwind; 0.08 (±0.02) s, which is similar to the value (0.10 s) obtained by Linthorne [14] in a study of competition results. However, the study by Hollings and colleagues might have obtained more accurate estimates for altitude and wind had it also considered the historical increase in performance (about 0.04 s) over the period of the study. Also, the accuracy of the method used by Hollings and colleagues would be strengthened if an analysis of elite female athletes was conducted and produced a value similar to that obtained for elite male athletes.

Several investigators have used mathematical models to calculate the time advantage of performing a 100-m sprint in Mexico City. For the force models developed by Dapena and Feltner [10], Mureika [11], and Behncke [9], the time advantages were 0.05 s, 0.08 s, and 0.10 s, respectively. For the power models developed by Arsac [8], Ward-Smith [6], and Péronnet and colleagues [7], the time advantages were 0.16 s, 0.17 s, and 0.18 s, respectively. Both types of model produced velocity-time curves that were similar to those for elite sprinters. However, the altitude predictions of the models are sensitive to the athlete’s effective drag area, which is only known to an accuracy of about 20% [21]. Also, Ward-Smith [21] suggested that a mathematical model of sprinting should include the effects of changes in aerodynamic drag on the angle of forward lean of the athlete’s body, as this is expected to affect the athlete’s ability to generate a forward propulsive force. In summary, results from mathematical modelling studies of the effect of altitude on sprint performance should be viewed with caution.

### 4.2. Implications for IAAF Rules

The breaking of national and world records is an important part of major athletics competitions, and some competitions offer substantial financial rewards to encourage athletes to set a record (e.g., $100,000 for a world record at the IAAF World Championships). For a performance to be recognized as a record, the performance must conform to specific regulations concerning the conduct of the event, the method of measuring the performance, the dimensions and properties of the track, the equipment used by the athlete, and doping control [1]. A fair system of recognizing record performances requires precise quantitative information on the effects of environmental conditions so that limits can be placed on the allowable range of conditions. At present, the only environmental factor that is considered when recognizing a record performance is the strength of the wind in the direction of running. In the 100-m sprint a performance will not be accepted if there is an assisting wind that averages more than 2 m/s.

Two important criteria when selecting the limits on an environmental condition are: (1) the limits should encompass the most commonly experienced conditions at the large majority of competition venues; and (2) the limits should eliminate only those performances that receive a substantial advantage relative to a performance achieved under the most common conditions. For the 100-m races at the Olympic Games between 1964 and 2012, about 94% had wind readings of between –2 and +2 m/s, and only 4% were not eligible for consideration for a record (*i.e.*, an assisting wind greater than +2 m/s). In the 100-m sprint a 2 m/s assisting wind produces a time advantage of about 0.10–0.12 s relative to a performance achieved with a zero wind [14]. If the wind limit were to be set substantially higher than 2 m/s, athletes would be unlikely to set a record under the most common wind conditions.

A similar reasoning can be applied to the selection of the limits on the altitude of a competition venue. About 75% of the Earth’s land surface is at an altitude of less than 1000 m [22], as are the vast majority of athletics competition venues [5]. If we assume the shape of the relationship between altitude and time advantage proposed by Arsac [8], the present study indicates that a time advantage of 0.10 s (equivalent to that from a 2 m/s assisting wind) is produced by an altitude of about 1000 m. Therefore, one can argue for an upper limit of about 1000 m when recognizing record performances. This proposed limit is the same as that already used by many athletics statisticians, who recognize the beneficial effect of high altitude by a listing sprinting and jumping performances achieved at venues above 1000 m as ‘altitude-assisted’ [5].

In addition to the 100-m sprint, the IAAF has a limit on wind assistance for the 200 m, 100 m hurdles, 110 m hurdles, long jump, and triple jump. The wind assistance limit is the same (2 m/s) for all these events, and it would be simplest if any altitude limit was also the same for all affected events. However, the present study of the effect of altitude was restricted to the 100-m sprint because this is the only event in which there is a reliable wind-correction curve [14]. Before studies similar to the present one can be conducted to determine of the effect of altitude on the other events, there needs to be reliable wind-correction equations for these events.

## 5. Conclusions

The time advantage for 100-m sprinters when competing at the 1968 Mexico City Olympic Games was 0.19 (±0.02) s for men and 0.21 (±0.05) s for women. This indicates that 100-m sprinters derive a substantial performance advantage when competing at a high-altitude venue. It is inferred that an altitude of 1000 m provides an advantage that is equivalent to a 2 m/s assisting wind (0.10 s). Therefore, the altitude of the competition venue should be considered when recognizing record performances in the 100-m sprint.
